# Supplementary material for: Information diffusion backbones in temporal networks
Source: Sci Rep. 2019 May 1;9:6798. doi: 10.1038/s41598-019-43029-5 (PMC6494818; doi:10.1038/s41598-019-43029-5)
Supplement: Supplementary file 1 — supplementary information [file 41598_2019_43029_MOESM1_ESM.pdf]

Supplementary Information for

Information diffusion backbones in temporal networks

Authors and affiliations

Xiu-Xiu Zhan[1], Alan Hanjalic[1], Huijuan Wang[1]*

March 1, 2019

[1]Faculty of Electrical Engineering, Mathematics, and Computer Science, Delft University of Technology, Mekelweg 4, Delft, The Netherlands, 2628 CD.

APPENDIX A: Data Description

Table S1: The lengths of the observation time window that we choose based on the average prevalence ρ when $\beta = 1$. For instance, $T_{90\%}$ represents the time when the prevalence reaches $\rho = 90\%$.

$Network$	$T_{90\%}$	$T_{80\%}$	$T_{70\%}$	$T_{60\%}$	$T_{50\%}$	$T_{40\%}$	$T_{30\%}$	$T_{20\%}$	$T_{10\%}$
RM	3325	1482	1278	987	257	133	111	34	5
HT2009	2394	2131	1575	1154	790	568	439	377	332
HS2011	1903	1177	1152	1001	805	447	425	396	47
HS2012	3915	2680	1907	1481	1109	1043	925	675	403
HS2013	1253	583	406	395	369	236	195	113	50
PS	997	510	378	359	347	323	287	276	136
WP	3328	2186	1538	1133	832	708	400	320	218
ME	27189	5096	1885	1735	1387	731	461	285	168
EEU	160710	134342	67883	27531	15792	8100	4047	2348	1490
Haggle	/	/	15640	14229	12668	12440	9523	8416	3293
Infectious	/	/	/	/	1062	955	751	553	410
DNC	/	/	/	18680	17712	14918	11420	7817	3860
Collegemsg	/	/	54493	46419	41663	33889	26018	17367	9747

APPENDIX B: Number of iterations to compute the backbone

We explore whether $h = 100$ iterations is sufficient to get a representative backbone when $0 < \beta < 1$. Given the temporal network and β, we first construct the diffusion backbones by choosing the number of iterations as $50, 100, 200, 300, 400, 500$, and then we compute the overlap r between the backbone obtained as the average of 100 iterations with the backbones obtained as the average of $50, 200, 300, 400, 500$ iterations, respectively. The overlap r is defined the same as Eq. (2). As the complexity of computing backbones is high, we consider a large number of networks but not all. Figure S1(d-f) shows the ratio of links in the observed $G_B(\beta)$ to $|\mathcal{L}_W|$ slightly increases with the increase of h. The overlap r is in general high, above 0.95 (Figure S1(a-c)). These observations support that we could obtain a relatively representative backbone as the average of 100 realizations of the backbone constructions. In addition, the slightly increase of link ratio also supports that the observed topology of $G_B(\beta)(0 < \beta < 1)$ is approaching G_W when the iteration times h is large enough.

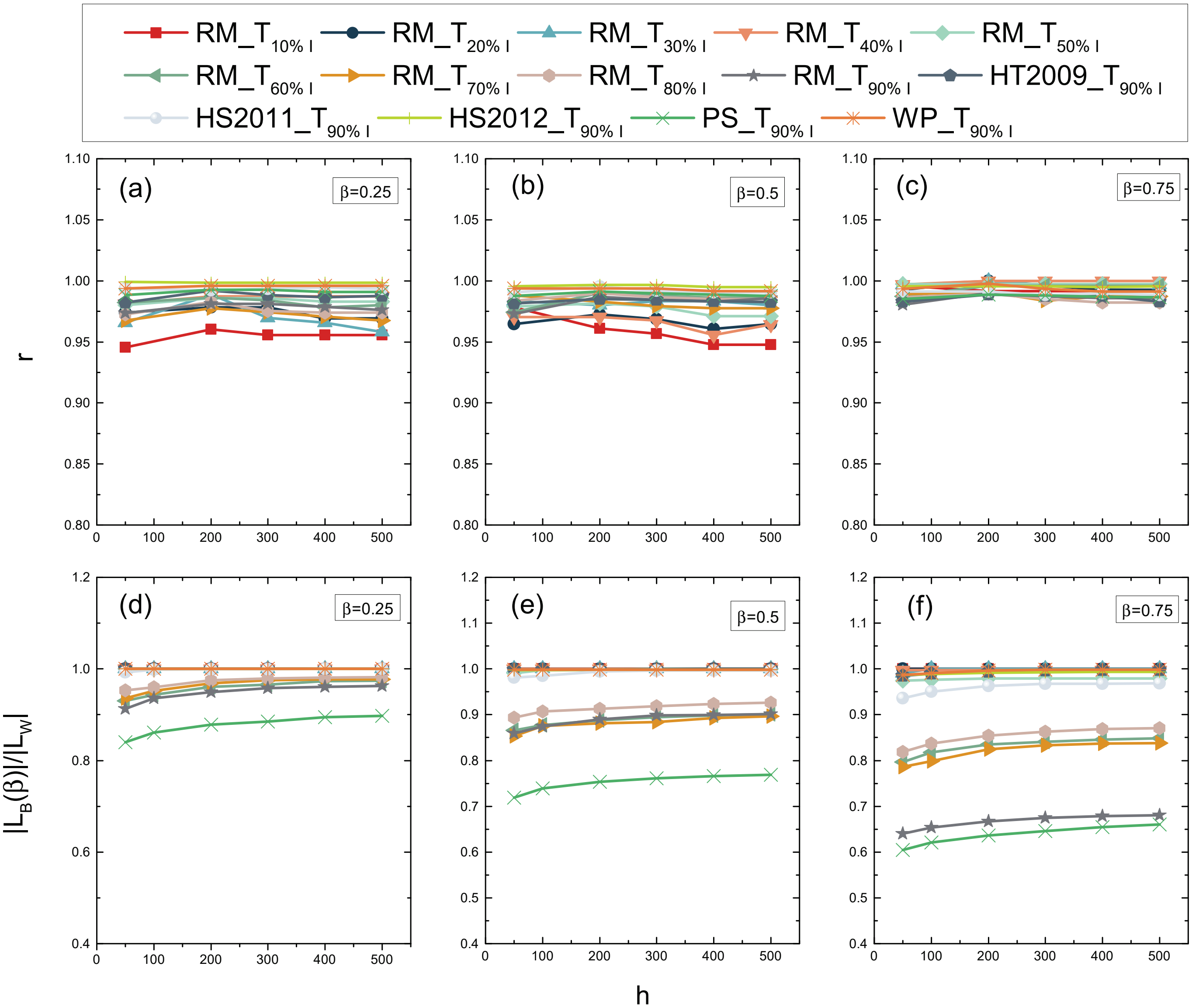

Figure S1: (a-c) Overlap r between backbone obtained from 100 iterations with the backbones obtained from $h = 50, 200, 300, 400, 500$ iterations on different temporal networks. (d-f) The ratio of links in the observed $G_B(\beta)$ to $|\mathcal{L}_W|$ in the backbones as a function of the number of iterations.

APPENDIX C: Relationship between backbones

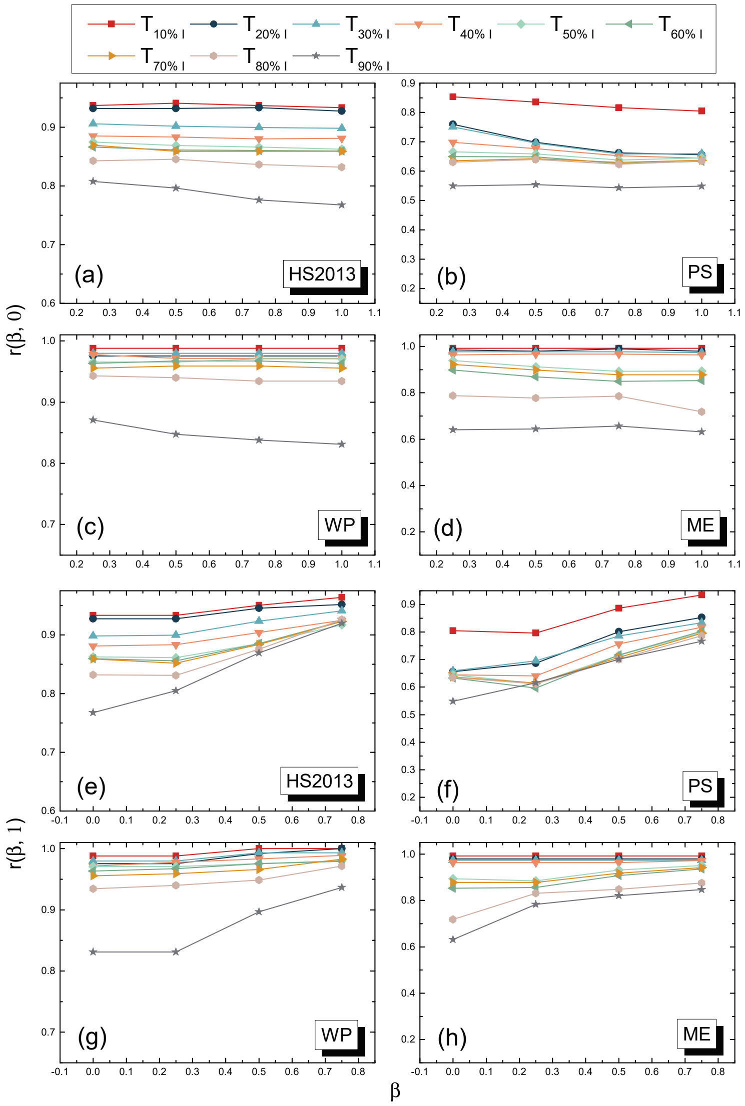

Figure S2: (a-d) Overlap $r(\beta, 0)$ between $G_B(\beta)$ and $G_B(0)$ as a function of β in (sub)networks derived from dataset $HS2013$, PS, WP and ME; (e-h) Overlap $r(\beta, 1)$ between $G_B(\beta)$ and $G_B(1)$ as a function of β in (sub)networks derived from dataset $HS2013$, PS, WP and ME. Diffusion backbones ($0 < \beta < 1$) are obtained from 100 iterations.

APPENDIX D: Identification of links in $G_B(1)$

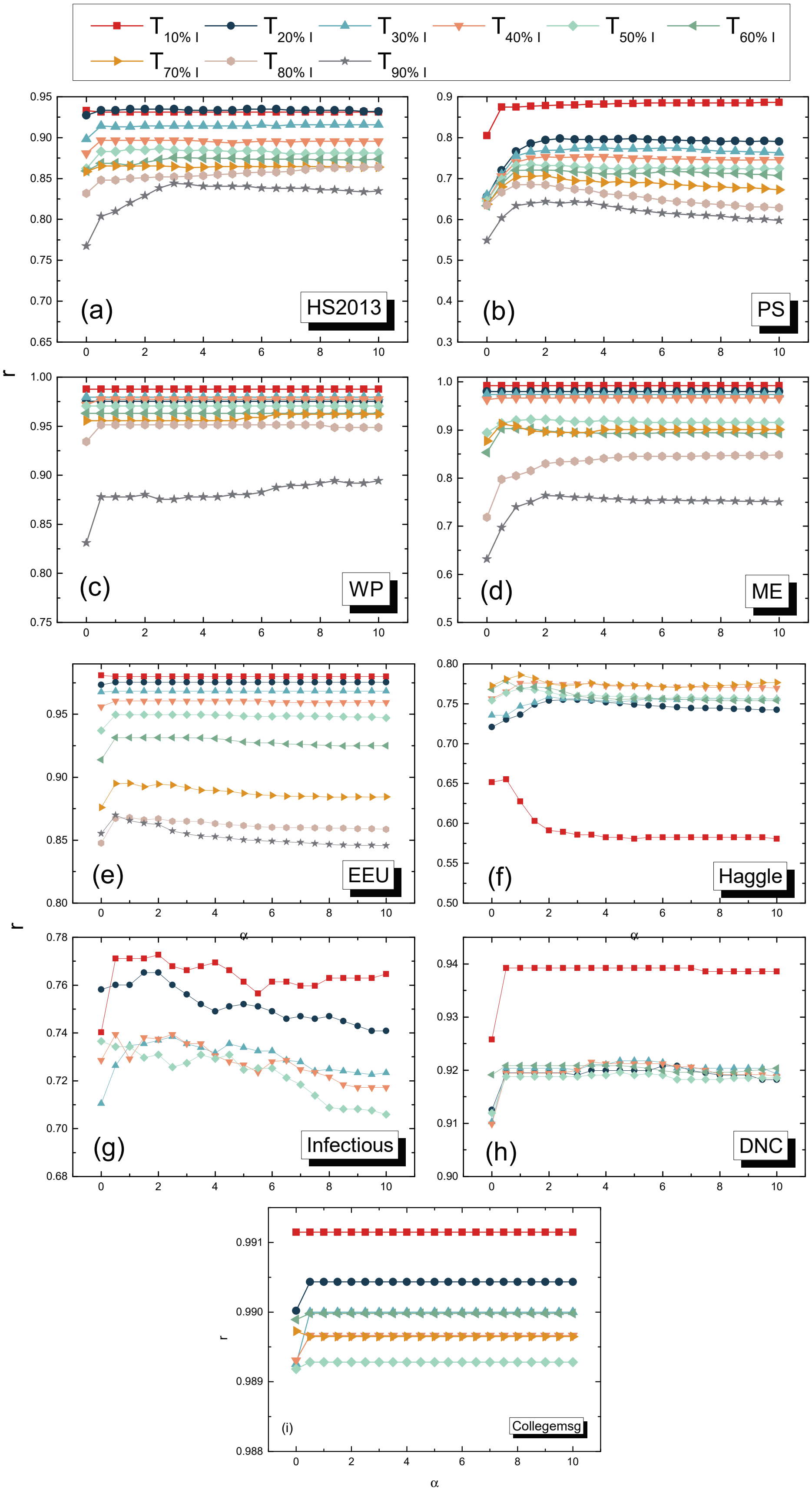

Figure S3: The quality of identifying links in $G_B(1)$ by using the time-scaled weight $\phi_{jk}(\alpha)$ as a function of α in temporal networks derived from datasets (a) $HS2013$; (b) PS; (c) WP; (d) ME; (e)EEU; (f) $Haggle$; (g) $Infectious$; (h) DNC; (i) $Collegemsg$.

APPENDIX E: Degree correlation between G_W and $G_B(1)$

Table S2: Pearson correlation coefficient $P(G_W, G_B(1))$ between node degree in G_W and $G_B(1)$ in all the networks.

$Network$	$T_{90\%}$	$T_{80\%}$	$T_{70\%}$	$T_{60\%}$	$T_{50\%}$	$T_{40\%}$	$T_{30\%}$	$T_{20\%}$	$T_{10\%}$
RM	0.8491	0.8672	0.8380	0.8461	0.9775	0.9908	0.9930	0.9992	1
HT2009	0.9665	0.9744	0.9831	0.9830	0.9911	0.9924	0.9956	0.9915	0.9829
HS2011	0.9352	0.9318	0.9256	0.9673	0.9558	0.9549	0.9746	0.9703	0.9722
HS2012	0.9656	0.9763	0.9829	0.9856	0.9875	0.9873	0.9849	0.9853	0.9866
HS2013	0.9368	0.9634	0.9717	0.9739	0.9747	0.9791	0.9784	0.9865	0.9857
PS	0.7022	0.7606	0.7836	0.7996	0.8180	0.8195	0.8033	0.7808	0.9051
WP	0.9422	0.9899	0.9914	0.9934	0.9929	0.9938	0.9956	0.9937	0.9975
ME	0.7624	0.9442	0.9892	0.9265	0.9877	0.9967	0.9977	0.9981	0.9990
EEU	0.9913	0.9909	0.9920	0.9936	0.9936	0.9965	0.9968	0.9965	0.9967
Haggle	/	/	0.9872	0.9859	0.9844	0.9842	0.9838	0.9815	0.9734
Infectious	/	/	/	/	0.9421	0.9447	0.9270	0.9347	0.9252
DNC	/	/	/	0.9967	0.9960	0.9950	0.9941	0.9933	0.9935
Collegemsg	/	/	0.9999	0.9999	0.9999	0.9998	0.9998	0.9998	0.9998
